# Integrated DNA methylation analysis identifies topographical and tumoral biomarkers in pilocytic astrocytomas

**DOI:** 10.18632/oncotarget.24480

**Published:** 2018-02-12

**Authors:** Manila Antonelli, Antonio Fadda, Eleonora Loi, Loredana Moi, Cesare Zavattari, Pia Sulas, Davide Gentilini, Cinzia Cameli, Elena Bacchelli, Manuela Badiali, Antonella Arcella, Isabella Morra, Felice Giangaspero, Patrizia Zavattari

**Affiliations:** ^1^ Department of Radiological, Oncological and Anatomo-Pathological Sciences, University Sapienza of Rome, Rome, Italy; ^2^ Unit of Biology and Genetics, Department of Biomedical Sciences, University of Cagliari, Cagliari, Italy; ^3^ Bone Marrow Transplantation Unit, Microcitemico Children's Hospital, Cagliari, Italy; ^4^ Independent Researcher, Machine Learning, Lucca, Italy; ^5^ Unit of Oncology and Molecular Pathology, Department of Biomedical Sciences, University of Cagliari, Cagliari, Italy; ^6^ Department of Brain and Behavioral Sciences, University of Pavia, Pavia, Italy; ^7^ Bioinformatics and Statistical Genomics Unit, Istituto Auxologico Italiano IRCCS, Cusano Milanino, Milan, Italy; ^8^ Department of Pharmacy and Biotechnology, University of Bologna, Bologna, Italy; ^9^ IRCCS Neuromed, Pozzilli, Italy; ^10^ Department of Pathology OIRM-S, Anna Hospital, A.O.U. City of Health and Science, Turin, Italy

**Keywords:** pilocytic astrocytomas, methylome alteration, topographic and tumor biomarkers, gene expression alteration, human methylation beadchips

## Abstract

Pilocytic astrocytoma (PA) is the most common glioma in pediatric patients and occurs in different locations. Chromosomal alterations are mostly located at chromosome 7q34 comprising the *BRAF* oncogene with consequent activation of the mitogen-activated protein kinase pathway. Although genetic and epigenetic alterations characterizing PA from different localizations have been reported, the role of epigenetic alterations in PA development is still not clear. The aim of this study was to investigate whether distinctive methylation patterns may define biologically relevant groups of PAs. Integrated DNA methylation analysis was performed on 20 PAs and 4 normal brain samples by Illumina Infinium HumanMethylation27 BeadChips.

We identified distinct methylation profiles characterizing PAs from different locations (infratentorial vs supratentorial) and tumors with onset before and after 3 years of age. These results suggest that PA may be related to the specific brain site where the tumor arises from region-specific cells of origin. We identified and validated *in silico* the methylation alterations of some CpG islands. Furthermore, we evaluated the expression levels of selected differentially methylated genes and identified two biomarkers, one, *IRX2*, related to the tumor localization and the other, *TOX2*, as tumoral biomarker.

## INTRODUCTION

Pilocytic astrocytoma (PA) is a pediatric low-grade glioma (pLGG) and the most common pediatric brain tumor, accounting about for 18% of all pediatric brain tumors and mostly affecting children between 5–15 years of age. It can arise anywhere in the CNS, but is most commonly localized in the cerebellum followed by the optic pathway/hypothalamic region [1]. It is classified as grade I by the World Health Organization (WHO), reflecting their slow growth and typically non-invasive behavior.

Pilocytic astrocytomas typically contain a BRAF fusion but occasionally a BRAF V600E mutation, RAF1 fusion, intragenic duplication of FGFR1, or other rarer alterations are present [2, 3].

Childhood pilocytic astrocytomas (PA) are low grade tumours with an excellent prognosis. However, PA can cause extensive morbidity due to local tumor expansion or therapy-related side effects and recurrence or progressive disease (PD), which occurs in up to 80% of patients, depending on location and extent of initial resection [4]. Therefore, new therapies are needed in order to specifically target the disease and improve the clinical course of these patients.

Epigenetic biomarkers represent a promising area of research, with DNA methylation having the potential to provide information regarding physiological and pathological status. Methylation signatures can be useful as specific and accurate biomarkers to assist with prognosis. The aims of the present work were to define biologically distinct groups of PA and possible relevant biomarkers through a global DNA methylation analysis over 27K CpG loci, and to asses the impact of methylation alterations on gene expression by qRT-PCR. We identified distinct methylation profiles characterizing PAs from different locations (infratentorial *vs* supratentorial) and tumors with onset before (≤ 3 yrs) and after (> 3 yrs) 3 years of age. Our study also identified *IRX2* as possible topographical biomarker and *TOX2* as tumoral biomarker.

## RESULTS

### Differential methylation analysis

We performed a global DNA methylation profiling using Illumina Infinium HumanMethylation27 BeadChips on 20 PAs and 4 normal brain samples from healthy individuals. We carried out a differential methylation analysis between tumoral and non-tumoral samples, by comparing the average beta signal obtained for each locus in the two subgroups. We found 919 CpG loci resulting hypermethylated in tumoral tissues at the nominal *p*-value threshold 5.0E-02 (differential score above 13). 640 of them were also significant with *p*-value below 1.0E-02, and 449 below 1.0E-03. On the other side, 1544 loci were significantly hypomethylated in cancer samples (*p*-value < 5.0E-02), of which 933 under 1.0E-02 and 609 under 1.0E-03.

Although the number of samples analyzed was quite small, we divided PA patients into two subgroups according to two selection criteria: topographic criterion and age of onset criterion in order to test whether these parameters may correlate with different methylation patterns. According to the topographic criterion, 50% of PAs (10/20) had a supratentorial localization and the other half of tumors (10/20) were located in the infratentorial region. The differential methylation analysis between the two clusters revealed 1931 CpG loci resulting hypermethylated in the supratentorial PAs at the nominal *p*-value threshold 5.0E-02. 1276 of them were also significant with *p*-values below 1.0E-02, and 859 below 1.0E-03. On the other side, 1381 loci were significantly hypomethylated in the supratentorial PAs (*p*-value < 5.0E-02), of which 843 under 1.0E-02 and 519 under 1.0E-03.

Next, we tested the hypothesis whether the age of onset correlated with the DNA methylation pattern. Since brain tumors in children under 3 years of age differ in clinical presentation and biological behavior from those in older patients, cases were grouped as follows: 45% of tumors (9/20) with onset before 3 years of age (≤3 yrs) and 55% of PAs (11/20) developed after 3 years of age (>3 yrs). The differential methylation analysis revealed 2645 CpG loci resulting hypermethylated in the >3 yrs subgroup, at the nominal *p*-value threshold 5.0E-02. 1506 of them were also significant with *p*-values below 1.0E-02, and 899 below 0.001. On the other side, 2312 loci were significantly hypomethylated in the >3 yrs subgroup (*p*-value < 5.0E-02), of which 1403 under 1.0E-02 and 828 under 1.0E-03.

**Figure 1:**
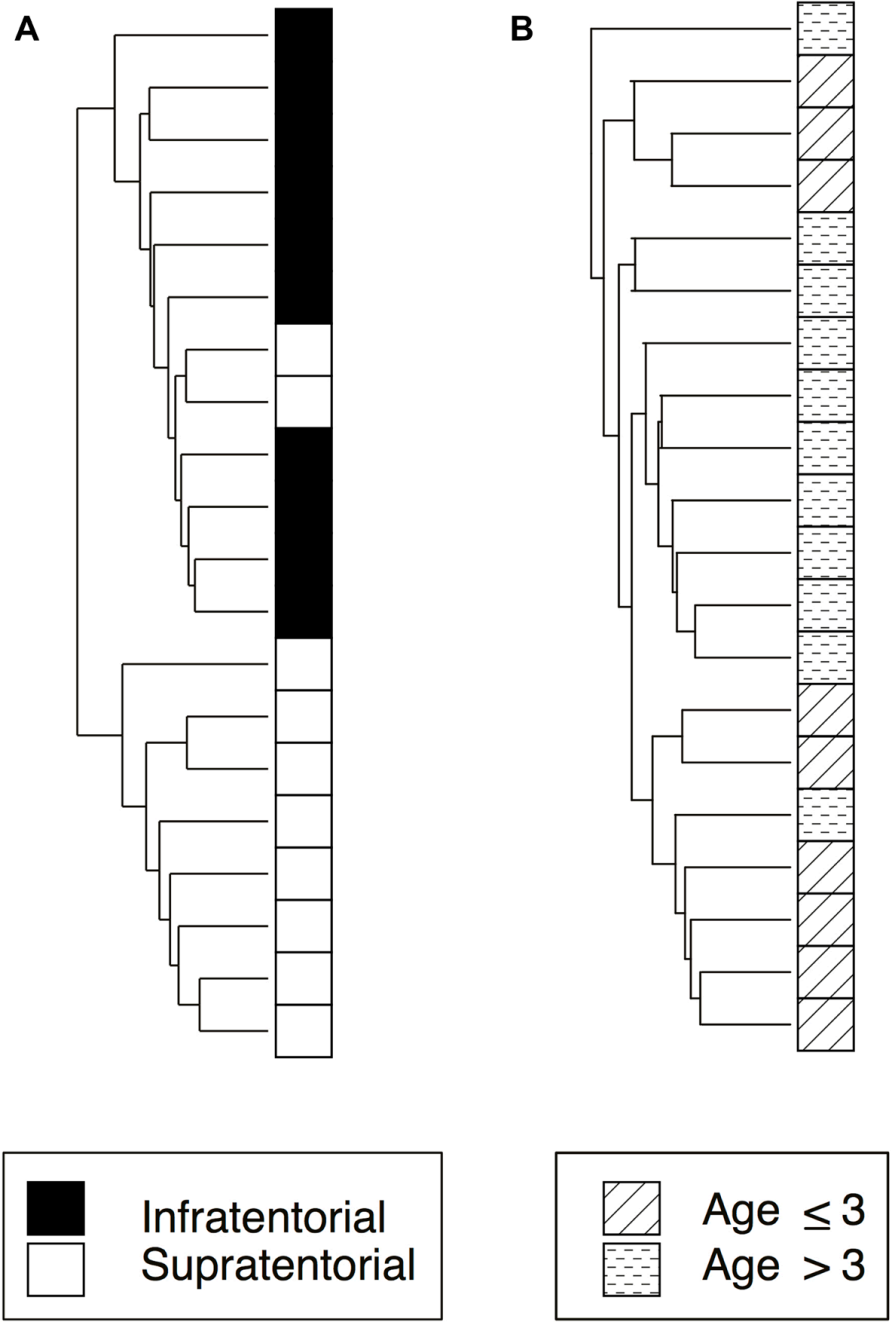
Unsupervised hierarchical clustering analysis of significantly differently methylated CpG Islands related to the two subgroups considered in the study: tumor location and age at onset (**A**) infratentorial and supratentorial samples (248 CpG Islands); (**B**) age at onset ≤ 3 or > 3 (360 CpG Islands).

The Unsupervised Hierarchical Clustering (UHC), performed by comparing the methylation value of each sample for each locus, demonstrates that the relevant methylated loci (*p*-value < 0.01) allow to distinguish two major clusters for each category, infratentorial *vs* supratentorial (Figure 1A) and age ≤ 3 *vs* age > 3 (Figure 1B) mostly reflecting the subgroups established *a priori*. In particular, PAs segregated clearer based on tumor location than age groups; in fact, only two supratentorial tumors clustered with the infratentorial PAs.

We conducted Ingenuity Pathway Analysis in order to classify genes associated with differentially methylated loci thus identifying pathways potentially involved in gliomas oncogenesis.

IPA revealed that genes associated to significantly differently methylated CpG loci in the case-control study (*p*-value < 1.0E-03) belong to the following networks: Gαi Signaling; RhoGDI Signaling; CMP-N-acetylneuraminate Biosynthesis I (Eukaryotes); cAMP-mediated signaling; Synaptic Long Term Depression. The analysis also suggested which cellular and molecular circuits were mainly affected, including: cancer mechanisms, neurological diseases, molecular transport, cellular growth and proliferation, cell cycle, DNA replication, recombination and repair, tissue development, cell to cell signaling and interaction, etc. As expected, the highest number of genes with altered methylation belongs to pathways involved in the development, growth and proliferation of cells and tissues, including the mechanisms of cell death, survival, and cancer. The involvement of these networks is supported by quite strong statistical evaluation (*p*-value < 2.0E-04).

Then, analyzing by IPA, the list of genes whose CpG loci are differentially methylated between supratentorial *vs* infratentorial tumors, we identified these pathways: cAMP-mediated signaling; G-Protein Coupled Receptor Signaling; LXR/RXR Activation; Maturity Onset Diabetes of Young (MODY) Signaling; FXR/RXR Activation.

Finally, by entering into IPA, the list of CpG loci differently methylated between ≤3 yrs and >3 yrs subgroups, the molecular pathways that appear to be most affected are: p53 Signaling; Intrinsic Prothrombin Activation Pathway; Sphingosine-1-phosphate Signaling; α-tocopherol Degradation; Role of IL-17A in Psoriasis.

### Calibration of an integrated DNA methylation analysis approach: the *EN2* case

**Figure 2:**
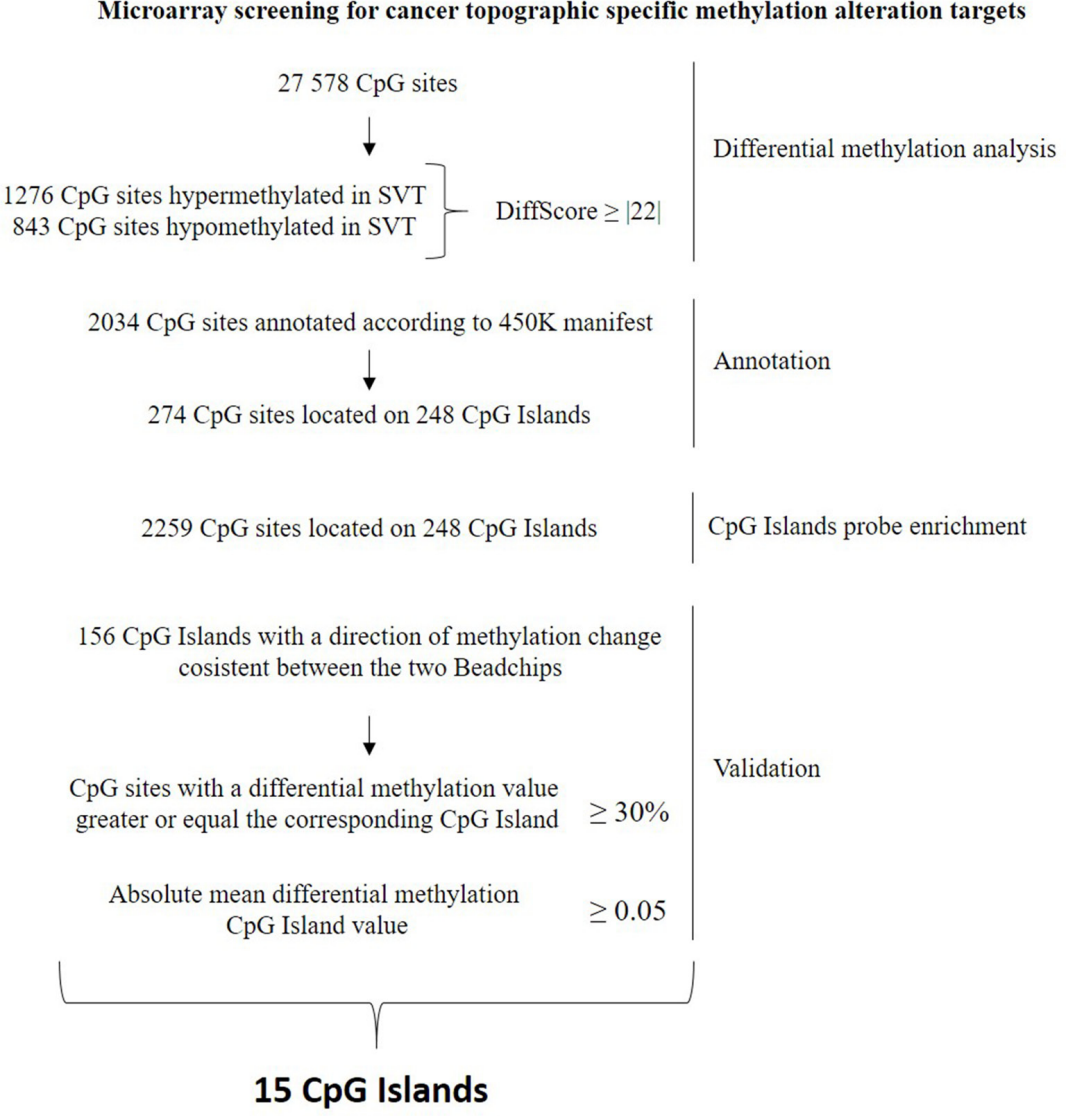
Flowchart describing the selection, annotation, probe enrichment and validation of the methylation alterations relative to the Supratentorial/Infratentorial differential methylation comparison

To validate and increase the robustness of HumanMethylation 27 data taking advantage of the wider epigenome coverage provided by the 450K array, we performed a probe enrichment (see methods and Figure 2) of our data using the 450K data obtained by Lambert *et al.* analyzing 62 PA samples (GEO repository of NCBI [GSE44684]) [5].

**Figure 3:**
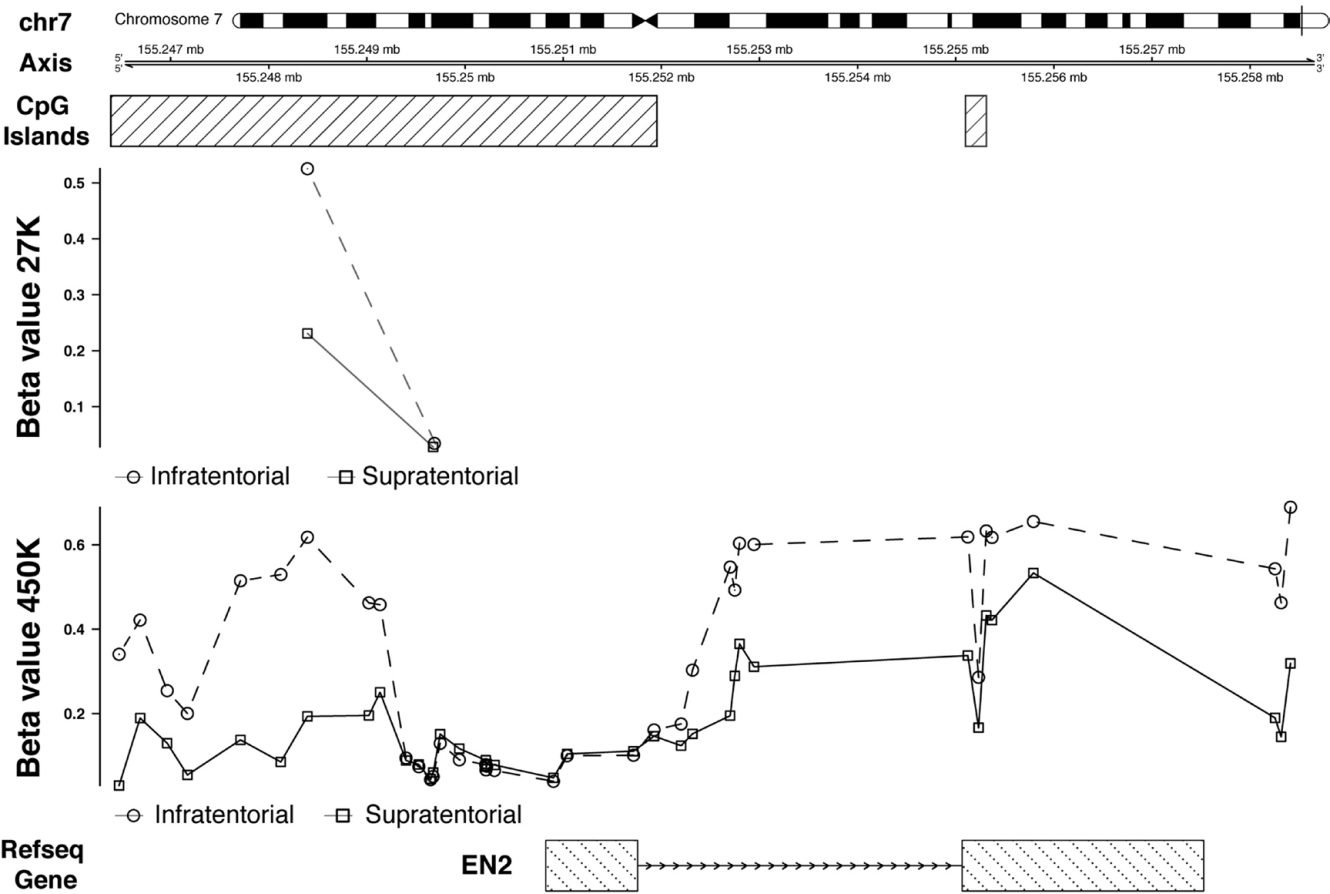
27K/450K enrichment and comparison CpG Islands found significantly altered in PAs analyzed in the present study (using the HumanMethylation27 beadchip), were compared and enriched *in silico* with PA data analyzed by Lambert and colleagues (HumanMethylation450K beadchip). Here is the example of the *EN2* gene.

First of all, we were able to validate the hypermethylation of *EN2*, key result in Lambert *et al.* [5]. In particular, as reported by Lambert *et al.* [5], this gene shows an extensive gene body hypermethylation, including the regions upstream and downstream the gene, except the region around the transcription start site (TSS) (Figure 3).

**Figure 4:**
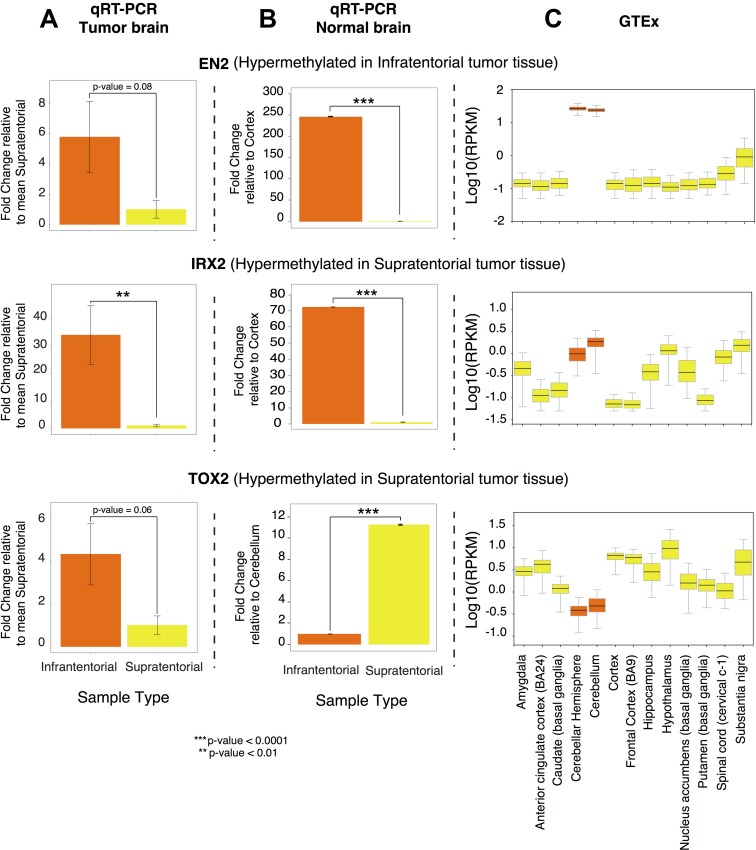
*EN2, IRX2, TOX2* gene expression profile (**A**) Differential expression obtained by qRT-PCR on the PA samples collected in the present study, calculated using the ΔΔCT method and expressed as fold change relative to the supratentorial mean expression value; (**B**) Differential expression obtained by qRT-PCR on commercially available normal brain RNA, calculated using the ΔΔCT method and expressed as fold change relative to the lowest expression value; (**C**) Gene expression in normal brain tissue, according to GTEx portal (Genotype-Tissue Expression portal). Expression value are shown in log10(RPKM) (Reads Per Kilobase of transcript per Million mapped reads), calculated from a gene model with isoforms collapsed to a single gene. In orange: supratentorial localization and related brain regions; in yellow: infratentorial localization and related brain regions.

To examine the influence of DNA methylation on gene expression, *EN2* expression analysis was assessed by qRT-PCR, revealing that its expression was higher in infratentorial than in supratentorial PAs by almost 6-fold (Figure 4A).

We compared the expression levels of *EN2* observed in our tumor samples with patterns of expression in commercially available human normal brain samples (Figure 4B) and in different normal brain sites using gene expression data retrieved from the GTExPortal, showing that its expression level in the tumoral samples is in line with that observed in the normal tissues (Figure 4C).

### Identification of novel biomarkers

Since our prominent results emerged from the differential methylation analysis between the two PA localizations, we focused our validation process only on these results.

The list of genes validated is reported in Supplementary Tables 1 and 2.

We focused on the genes *IRX2* and *TOX2* to verify whether a differential DNA methylation may correlate with different gene expression profiles between the two tumoral localizations. Due to the limited amount of RNA available from our samples, six genes were selected based on 27K absolute mean differential methylation value greater or equal to the 10%. The feasibility of the assay design, that guaranteed reproducibility and reliability of the gene expression data, led to the selection of *IRX2* and *TOX2* for subsequent qRT-PCR analysis.

**Figure 5:**
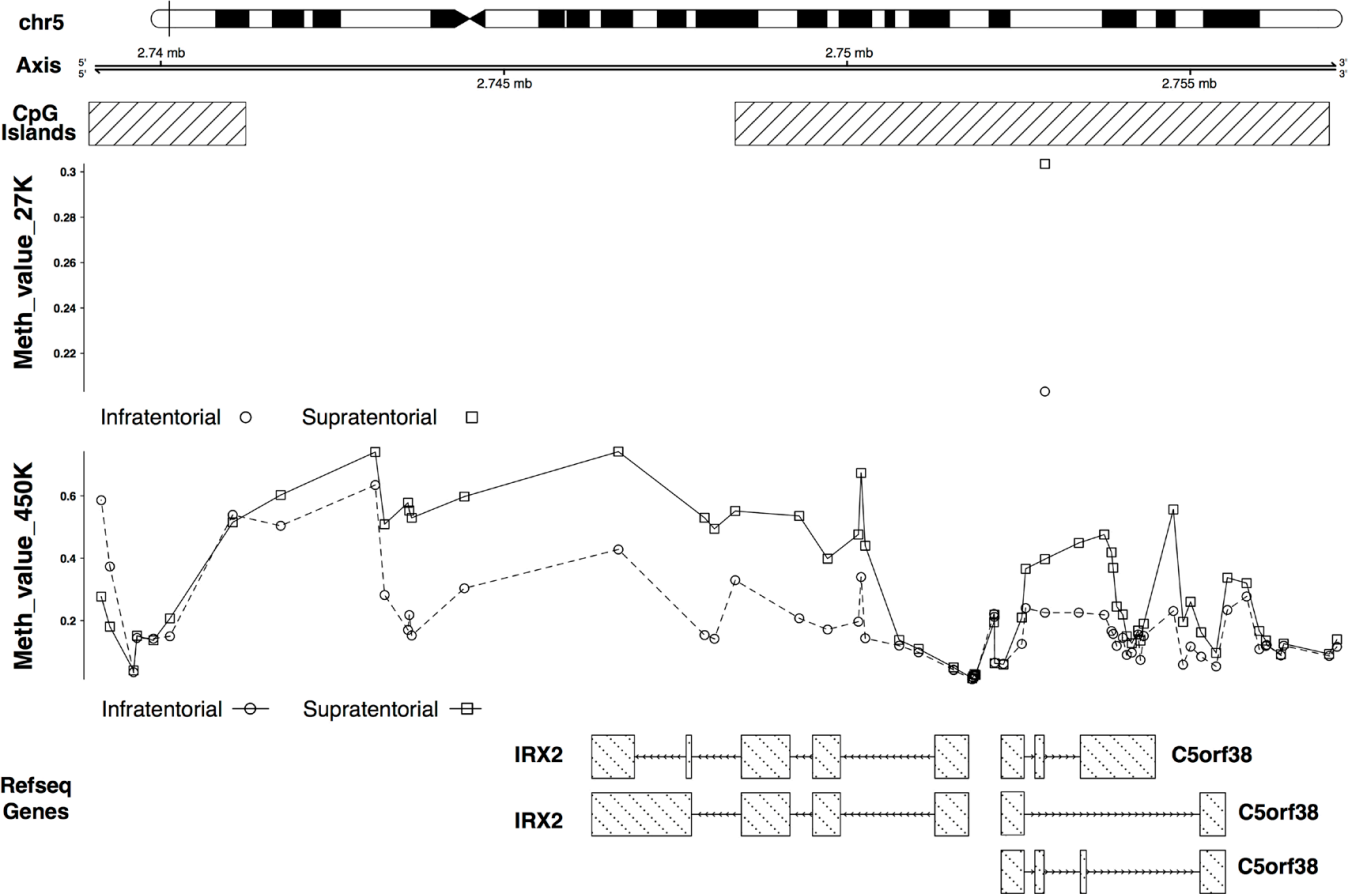
27K/450K enrichment and comparison CpG Islands found significantly altered in PAs analyzed in the present study (using the HumanMethylation27 beadchip), were compared and enriched *in silico* with PA data analyzed by Lambert and colleagues (HumanMethylation450K beadchip). Here is the example of the *IRX2* gene.

**Figure 6:**
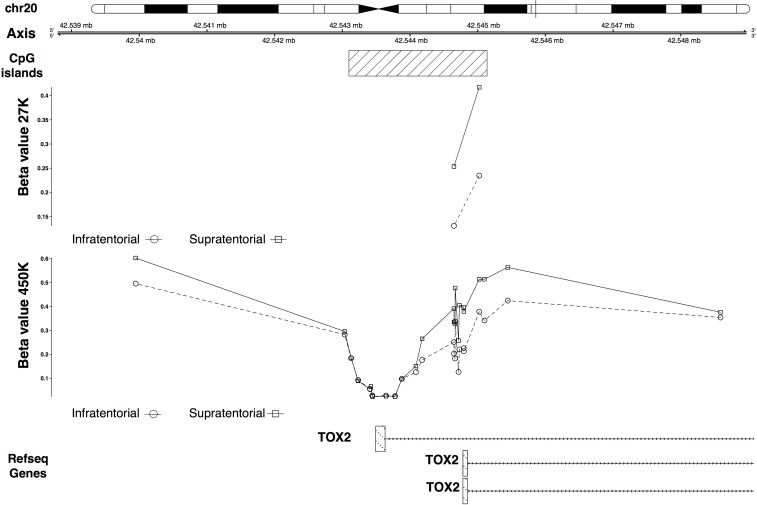
27K/450K enrichment and comparison CpG Islands found significantly altered in PAs analyzed in the present study (using the HumanMethylation27 beadchip), were compared and enriched *in silico* with PA data analyzed by Lambert and colleagues (HumanMethylation450K beadchip). Here is the example of the *TOX2* gene.

The *IRX2* and *TOX2* hypermethylation in the supratentorial PAs was validated (Figures 5, 6). In particular *IRX2* showed a hypermethylation, apart from the region around the TSS, of a CpG island that spans the promoter region and the gene body (Figure 5); while *TOX2* showed a promoter CpG island hypermethylation, except for the region around the TSS (Figure 6).

qRT-PCR revealed a significantly decreased expression of *IRX2* (Figure 4A) and almost significant of *TOX2* (Figure 4A) (by nearly 35 fold and by about 4 fold respectively) in supratentorial compared to infratentorial PAs.

*IRX2* and *TOX2* gene expression levels in tumor samples (Figure 4A) were compared to commercially available human normal brain samples (Figure 4B) and to GTEx expression data observed in different normal brain sites (Figure 4C). The expression level of *IRX2* in the tumor samples resulted in line with that observed in the normal brain sites. On the other hand, *TOX2* expression level in the PA samples is in contrast to the gene expression level observed in the normal brain sites.

### Prognostic value of the identified biomarkers

**Figure 7:**
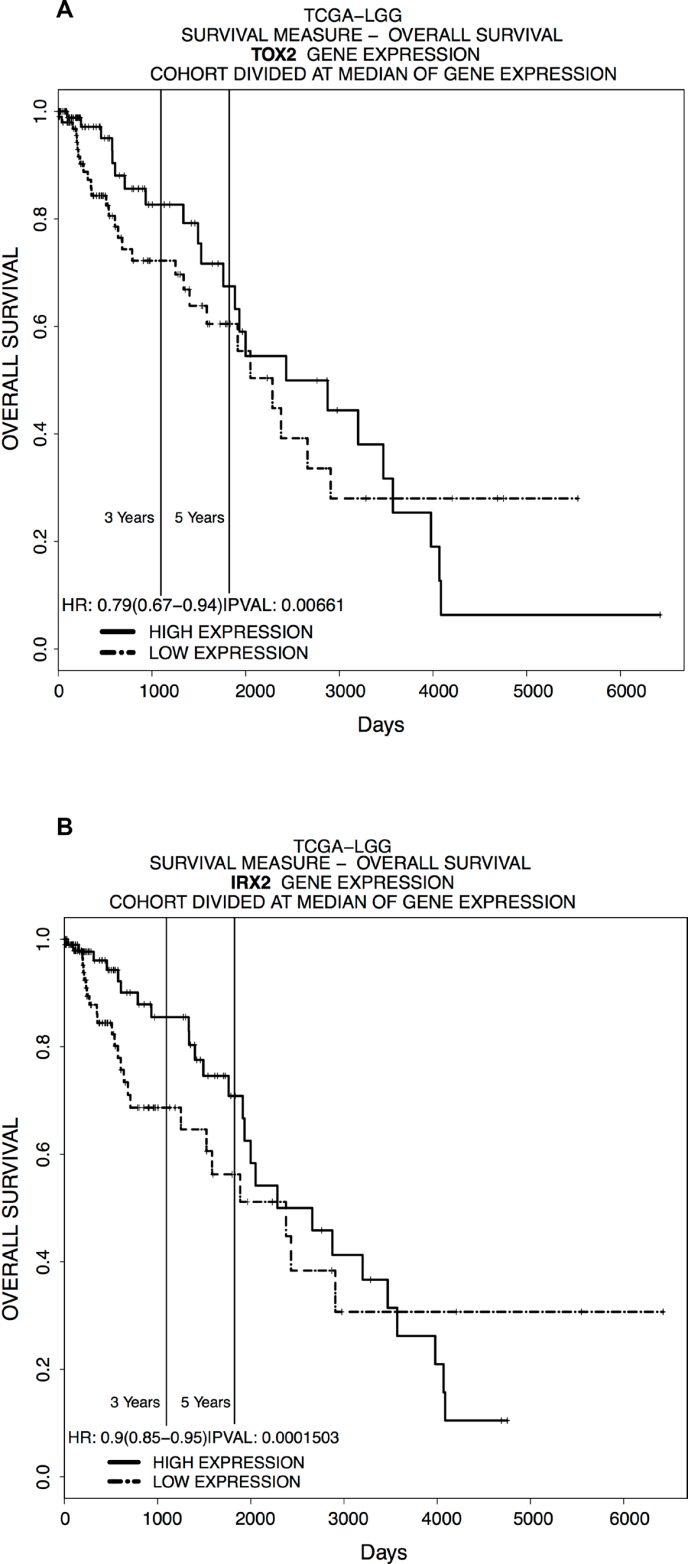
*In silico* LGG survival curves of patients classified by (**A**) *TOX2* and (**B**) *IRX2* expression in the indicated dataset [TCGA-LGG] (x-axis: survival time in days; y-axis: overall survival). Samples were divided into high and low expression groups bifurcating at median expression value for mRNA expression.

Since the results obtained showed a differential methylation and expression of *TOX2* and *IRX2* between supratentorial and infratentorial PAs, we decided to evaluate the putative prognostic implications of *TOX2* and *IRX2* expression using a web-tool called PROGgeneV2 [6] on *in silico* data. Patients were classified as high-expression or low-expression groups to generate Kaplan-Meier plots (Figure 7A, 7B) using gene expression data of adult LGG from TCGA [TCGA-LGG]. The results showed very significant differences in the survival rate between patients with different expression of *TOX2* (Figure 7A) and *IRX2* (Figure 7B).

## DISCUSSION

DNA methylation plays a key role during embryogenesis and differentiation. Alterations in DNA methylation have been linked to several human diseases including gliomas.

In this study we performed the following differential methylation analyses in PAs: 1) tumors *vs* normal brain, 2) ≤3yrs *vs* >3yrs subgroups and 3) infratentorial *vs* supratentorial. In addition, we performed *in silico* functional and interaction analyses of differently methylated loci using IPA software. The results obtained suggest an involvement of different pathways depending on the specific analysis. As expected, by comparing the methylation pattern of tumor DNA *vs* non-neoplastic brain cells, the cellular and molecular circuits mainly represented included mechanisms of cell death, survival, and tumorigenesis.

On the other hand, IPA identified inflammatory response mechanisms and immune cell trafficking as the top cellular and molecular networks related to PA topography i.e. infratentorial *vs* supratentorial.

It is noteworthy that p53 signaling results to be involved at a very significant level (*p*-value = 4.35E-06), with 21 altered genes on 95, in the age groups comparison. Although previous studies have reported a lower *TP53* mutations frequency in high grade glioma patients under the age of 3 years than older children, suggesting that there may be two distinct pathways involved in the tumorigenesis [7, 8], *TP53* mutations have been rarely reported in PA [9]. Similarly, in our study, none of the tumor samples showed immunohistochemical expression of p53, which when mutated, accumulates in the nucleus due to prolonged half-life.

Among the genes differentially methylated between PA patients younger and older than 3 years, *KL* gene resulted hypermethylated in the >3yo subgroup. Interestingly, the promoter of this gene has been found to be methylated and consequently downregulated in other tumors [10–14].

These results highlighted specific brain-region and age-related methylation patterns, suggesting that different molecular pathways may be involved in the pathogenesis of PA.

This finding was supported by the identification of distinct gene expression [5, 15–20] and methylation profiles [5, 20, 21] between tumors having different localizations. Sharma *et al.* [16] described a specific gene expression pattern discriminating infratentorial and supratentorial PAs. They also observed that these signatures are specific of tumoral and normal astrocytes or neuronal stem cells from the same brain region, suggesting that these molecular patterns may be connected to the specific brain site where the tumor arises. The differentially expressed genes identified by Sharma and colleagues [16] are implicated in the development of the forebrain and the hindbrain. One of these genes, *IRX2*, was found differentially expressed [16, 18, 20] and differentially methylated between infratentorial and supratentorial PAs in our and other studies [5, 20]. In particular, Jeyapalan *et al.* [20] identified an intergenic CpG site associated to *IRX2* that resulted to be differentially methylated between the two tumor groups; whereas we detected a differential methylation of a CpG island that spans the promoter region and part of the gene body of *IRX2*. In our sample set, *IRX2* gene showed a hypermethylation and an expected downregulation in supratentorial PAs compared to the infratentorial PAs (Figure 4A), confirming the gene expression profile observed by the other studies [16, 18, 20]. The expression level of this gene was in line with that observed in the normal tissues (Figure 4B and Figure 4C), suggesting that its downregulation is probably more of a reflection of where the tumor develops rather than the onset of PA *per se*. In fact, IRX2 is implied in vertebrate embryos development and is expressed in the developing hindbrain. During normal development DNA methylation targets genes that are already repressed in order to facilitate gene repression instead of directly cause gene silencing [22]. Our results support the evidence that DNA methylation in cancer occurs in normally downregulated genes [23–26]. Hence, the expression of some genes seemed to be finely regulated by DNA methylation and, in agreement to the model proposed by Timp *et al.*, these epigenetic alterations may disrupt the functions of genes that regulate the epigenome itself [27].

The possible involvement of *IRX2* in cancer development is also supported by the fact that IRX2 may act as a metastasis suppressing protein since its low expression has been found to be correlated with less differentiated and more aggressive breast cancer tumors. Furthermore, it has been revealed that it may be a repressor of chemokines expression, thus its low expression levels may lead to a sustained chemokines expression and a consequent mobilization of tumoral cells [28].

Other members of the Iroquois (IRO/IRX) family, *IRX1*, *IRX3* and *IRX5* have been found differentially methylated [5, 20] and/or expressed [5, 16, 18, 20] between supratentorial and infratentorial PAs, suggesting that these genes may have an important role in specific location cancer development.

Another developmental gene, *EN2*, showed a differential methylation and expression between supratentorial and infratentorial PAs.

*EN2* resulted to be hypermethylated and upregulated in the infratentorial PAs (Figure 4A). This result is in line with an unusual positive correlation, evidenced by Lambert *et al.*, [5] between non-TSS based hypermethylation and gene expression (i.e: high methylation values- upregulation; low methylation values–downregulation), of genes showing a differential methylation within the gene body and/or in regions upstream/downstream (within 10 kb) of the genes.

Interestingly, the expression levels of *EN2* in the normal cerebellar hemisphere is the highest among all brain sites according to commercially available RNA from human normal brain samples (Figure 4B) and to GTEx gene expression annotations (Figure 4C). EN2 is involved in the control of pattern formation during development of the central nervous system.

An interesting result of our study regards *TOX2* gene that showed a differential methylation between supratentorial and infratentorial tumors with the exception of the region around the TSS of the longest isoform showing no significant difference between the two localizations. *TOX2* was hypermethylated in supratentorial PAs and qRT-PCR revealed that it was also downregulated in this subgroup than infratentorial PAs (Figure 4A). This condition is in contrast to the gene expression level observed in the normal tissue according to commercially available RNA from human normal brain samples (Figure 4B) and to GTEx expression annotations (Figure 4C), suggesting its potential role as a tumoral biomarker.

This gene was found to be frequently hypermethylated in the 5′-end in a series of astrocytomas of different grades, including PA (grade I) [29]. Furthermore, by analyzing *in silico* methylation data from glioblastomas (GSE19391), we have been able to confirm that the methylation alteration near *TOX2* gene is also present in high grade gliomas [29]. The methylation difference between glioblastomas and non-tumor tissues is about 40%, so even higher than that observed in PAs, both by our group and by Lambert *et al.* [5]. The *in silico* analysis of these high grade gliomas also allowed us to point out that *TOX2* hypermethylation in glioblastomas seems to be independent from tumor localization and is present in both pediatric and adult age.

*TOX2* was also found to be hypermethylated and consequently downregulated in breast and lung cancer [30]. This study showed that *TOX2* knockdown modulates different molecular pathways such as tissue remodeling, inflammatory response and cell differentiation. The molecular function of this gene is still unknown although it has been suggested that it might be a putative transcriptional activator involved in the hypothalamo-pituitary-gonadal system such as the TOX2 ortholog (GCX-1), identified from a rat granulosa cell cDNA library [31].

Tessema and colleagues [30] have identified two transcript variants: var.5 and var.6. According to the aminoacid sequences deduced from these two transcripts, the isoform called var.5 presents a HMG box domain showing 100% homology with the HMG box domain of GCX-1, suggesting that TOX2 may be a transcriptional activator in humans. On the other hand, var.6 does not encode for the HMG box domain, thus this TOX2 isoform does not bind the DNA and may be a negative competitor of the other variants. In our qRT-PCR experiments, we designed an assay able to amplify all isoforms. It is known that most genes have downstream start sites within the gene bodies of the transcriptional units of the upstream promoters and methylation may be a mechanism for controlling alternative promoter usage [32]. Thus, it would be interesting to individually quantify the different isoforms and to study the possible impact of methylation alterations on alternative isoforms expression.

To explore whether *IRX2* and *TOX2* may also have an impact on tumor prognosis, we performed a survival analysis using a web-tool called PROGgeneV2. This tool allows to select a dataset containing gene expression data for a gene of interest. Since the unavailability of a specific dataset for pediatric PAs, the Kaplan-Meier plots (Figure 7A, 7B) were generated using gene expression data of adult LGG from TCGA [TCGA-LGG]. The results showed a significant difference in the survival rate between patients with different expression of *TOX2* (Figure 7A) and *IRX2* (Figure 7B), suggesting that these genes may be important predictive biomarkers of survival also in adult patients affected by different types of LGG gliomas.

Interestingly, gene ontology annotations related to the above mentioned genes include: sequence-specific DNA binding (EN2, and IRX2), chromatin DNA binding (TOX2), RNA polymerase II transcription factor binding (TOX2). EN2 and IRX2 contain a conserved DNA sequence encoding a homeodomain that specifically binds to DNA motifs. It has been established that the inhibition of homeobox genes by promoter CpGs island hypermethylation contributes to the inactivation of regulatory or DNA repair genes, concurring to tumorigenesis. Moreover, it has been recognized that homeobox hypermethylated genes in human neoplasms [33–38], including PA [5] overlapped with known Polycomb targets. Polycomb group proteins form multi-protein complexes that dynamically alter chromatin structure by modifying specific residues in histone tails and recruit DNA methyltransferases methylating DNA [39]. Polycomb-mediated repression is a principal mechanism by which *HOX* gene expression is tightly regulated during development [40]. Besides, Reddington *et al.* [41] speculate that DNA methylome reprogramming in cancer leads to an altered Polycomb binding landscape influencing gene expression regulation.

In conclusion, although we still do not functionally validate any of the identified genes as potentially relevant to the biology of PA, also because of the difficulty in culturing these lesions successfully, our results strongly suggest that PA may be related to the specific brain site where the tumor arises from region-specific cells of origin. Moreover, we found a specific topographical biomarkers (*IRX2)* confirming that different PAs segregate by tumor location. More interestingly, we identified *TOX2* as a promising tumor biomarker, suggesting its possible role in gliomas development, with a differential expression in PAs with different brain localization and a more pronounced hypermethylation in glioblastomas. TOX2 is involved in the control of cell apoptosis, growth, metastasis and DNA repair and is frequently deregulated in a variety of human malignances, however the functional role in tumors remain unspecified, and needed for future investigations.

DNA methylation biomarkers with diagnostic, prognostic and predictive power have great potential to contribute to personalized medicine throughout life.

The use of appropriate DNA methylation biomarker panels will prove beneficial where the disease phenotype is quite heterogeneous. It is also expected that the genetic component of disease will be further revealed, which will subsequently allow the strengthening of biomarker panels by combining genetic and DNA methylation biomarkers.

## METHODS

### Tumor specimens

Frozen tissue of twenty cases (*n* = 20) of pediatric (≤18 y) pilocytic astrocytomas were collected as part of the Italian National Program of Centralization of Pediatric Brain Tumor.

Frozen tissue samples from temporal lobes of adult healthy individuals (*n* = 4) submitted to epilepsy routine examination, were also collected.

The tumor samples were collected fresh at the time of surgery. Portions of resected tumors were snap frozen in liquid nitrogen and stored at –80° C until use, and the rest of the tissue was formalin fixed and paraffin embedded for routine histopathology. Haematoxylin-eosin stained sections of each case were reviewed carefully before they were selected for DNA extraction. Tumor areas from these specimens had been carefully selected by a pathologist to contain 60–90% neoplastic cells. This procedure allow us to evaluate difference in methylation between each case without surrounding normal tissue.

Clinicopathological data was available for all cases, including tumor location (Table 1).

**Table 1:** Sample description

*N*	Diagnosis	Tumor location	Sex	Age
1	PA	Cerebellum (I)	M	17
2	PA	Cerebellum (I)	F	7
3	PA	Cerebellum (I)	M	12
4	PA	Frontal lobe (S)	F	2
5	PA	Frontal lobe (S)	M	1
6	PA	Optic chiasm (S)	M	1
7	PA	Cerebellum (I)	F	13
8	PA	Cerebral hemisphere (S)	F	7
9	PA	Cerebellum (I)	M	11
10	PA	Optic chiasm (S)	M	2
11	PA	Cerebellum (I)	F	9
12	PA	Temporal lobe (S)	F	10
13	PA	Cerebellum (I)	F	3
14	PA	Cerebellum (I)	M	11
15	PA	Hypothalamus (S)	M	5
16	PA	Cerebellum (I)	M	3
17	PA	Cerebellum (I)	M	5
18	PA	Hypothalamus (S)	M	3
19	PA	Frontal lobe (S)	F	3
20	PA	Hypothalamus (S)	F	1

PA: pilocytic astrocytomas.

I: infratentorial.

S: supratentorial.

Only the samples with a tumor cell content estimated to be ≥70% from histopathological assessment were included for molecular analysis.

### Ethics approval and consent to participate

All procedures performed in studies involving human participants were in accordance with the ethical standards of the institutional and/or national research committee and with the 1964 Helsinki declaration and its later amendments or comparable ethical standards.

Informed consent was obtained from all individual participants included in the study.

### DNA samples purification

To isolate DNA from fresh frozen tissue, we used a commercial kit, called “DNeasy Blood & Tissue Kit” from Qiagen. DNA samples were analyzed quantitatively and qualitatively through spectrophotometric reading, using NanoDrop, fluorometric reading, by using PicoGreen as DNA intercalator, and by electrophoresis in a 0.8% agarose gel.

### Illumina infinium HumanMethylation27 BeadChips

20 PA samples and 4 control samples were analyzed using the Illumina Infinium HumanMethylation27 BeadChips according to the manufacturer’s instructions (Illumina, San Diego, USA Part#11322371 Rev. A).

### DNA methylation analysis

Methylation data were visualized and analyzed using the GenomeStudio^®^ software (Illumina). None of the samples were excluded following quality control steps assessed by bisulfite conversion, extension, staining, hybridization, target removal, negative and non-polymorphic control probes. Methylation levels [beta values (β)] were estimated as the ratio of signal intensity of the methylated alleles to the sum of methylated and unmethylated intensity signals of the alleles. The β values vary from 0 (no methylation) to 1 (100% methylation). CpG sites with a detection *p*-value > 0.05 were removed from analysis. Differential methylation levels (Δβ) between the groups of interest were calculated with the Illumina Custom model, as implemented in the Illumina GenomeStudio software, and DiffScores were computed. For a *p*-value of 0.05, DiffScore = ± 13; For a *p*-value of 0.01, DiffScore = ± 22; For a *p*-value of 0.001, DiffScore = ± 33. To account for multiple testing, the Illumina Custom Error Model with the False Discovery Rate (FDR) corrections was applied; we ran 1000 permutations and included FDR up to 20%.

The differential methylation probes were selected based on a DiffScore ≥ |22| and annotated according to the HumanMethylation450 (450K) manifest. The annotated probes on sex chromosome were excluded and only those present on a CpG Island were used for the subsequent analysis.

The corresponding CpG Islands were searched through the GSE44684 dataset [5] annotated according to the 450K, to perform the probe enrichment. This method (Figure 2) takes advantage of the wider coverage provided by the 450K to increase the number of probes for each significantly differently methylated CpG islands found with the HumanMethylation27 BeadChips. This allows, at the same time, an increase of the robustness of HumanMethylation27 results and their validation. The validation of our HumanMethylation27 results, based on the 450K data, was performed selecting those CpG Islands whose direction of methylation change was consistent between the two BeadChips. Furthermore, we established to select the CpG islands for which the percentage of probes, whose differential methylation value was greater or equal to the corresponding CpG island differential methylation mean value, was greater or equal to 30%. Only those CpG islands, with a mean absolute differential methylation value ≥ 0.05 and with at least 3 probes were considered. The list of genes validated is reported in Supplementary Tables 1 and 2.

The database NCBI Gene Expression Omnibus (GEO) portal (http://www.ncbi.nlm.nih.gov/geo/) was used to retrieve the *in silico* glioblastoma dataset under accession numbers GSE19391. For each CpG sites interrogated in the region of interest, processed data were used.

### Bioinformatics

The Gene Set Enrichment Analysis (GSEA) of the significant differently methylated loci were conducted by the Ingenuity Pathway Analysis (IPA) Software (Ingenuity Systems, Redwood City, CA, USA; www.ingenuity.com) from which are derived the information on the pathways potentially involved.

Gene expression data of normal brain tissues were retrieved from the Genotype-Tissue Expression (GTEx) Project (http://www.GTExportal.org), which contains data from gene expression microarrays and RNA sequencing.

The analysis of PA survival was performed using the ProGgeneV2 Prognostic Database (http://www.compbio.iupui.edu/proggene/) [6]. The Kaplan-Meier survival plots were constructed on *in silico* data using low-grade glioma survival data from TCGA [TCGA-LGG].

### qRT-PCR

RNA extraction from 3 supratentorial and 11 infratentorial tumor PA tissue samples stored in RNA later was performed using the RNeasy Mini Kit (Qiagen, Germany) following manufacturer’s instructions. Pooled normal brain total RNAs from different human brain areas were purchased from Clontech, in particular: Human Brain, Cerebellum Total RNA (636535) and Human Brain, Cerebral Cortex Total RNA (636561). Retro-transcription was performed starting from 1μg RNA/sample using the High Capacity Kit (Applied Biosystems, Carlsbad, CA, USA). Gene expression, conducted using ABI Prism 7500 Sequence Detection System (Applied Biosystems, Foster City, CA), was assessed by RT qPCR using SsoAdvanced^™^ Universal SYBR^®^ Green Supermix (Bio-Rad) for each gene tested and for the endogenous TFRC. Supplementary Table 3 summarized primers used in this study to conduct qRT-PCR. Gene expression data were analyzed using the ΔΔCT method. Statistical analyses were done using R function “*t*.test”.

## SUPPLEMENTARY MATERIALS TABLES








