# Supplementary material for: Integrated DNA methylation analysis identifies topographical and tumoral biomarkers in pilocytic astrocytomas
Source: Oncotarget. 2018 Feb 12;9(17):13807–21. doi: 10.18632/oncotarget.24480 (PMC5862617; doi:10.18632/oncotarget.24480)
Supplement: Supplementary file 1 [file oncotarget-09-13807-s001.pdf]

Integrated DNA methylation analysis identifies topographical and tumoral biomarkers in pilocytic astrocytomas

SUPPLEMENTARY MATERIALS

Supplementary Table 1: CpG Islands, and related genes, altered in the 27 K and validated in 450 K. See Supplementary_Table_1

Supplementary Table 2: Average methylation value for each sample at CpG Islands, and related genes, altered in the 27 K and validated in 450 K. See Supplementary_Table_2

Supplementary Table 3: Sequences of primers used for qRT-PCR. See Supplementary_Table_3
