# Supplementary material for: Integrated DNA methylation analysis identifies topographical and tumoral biomarkers in pilocytic astrocytomas
Source: Oncotarget. 2018 Feb 12;9(17):13807–21. doi: 10.18632/oncotarget.24480 (PMC5862617; doi:10.18632/oncotarget.24480)
Supplement: Supplementary file 2 [file oncotarget-09-13807-s002.docx]

**Supplementary Table 1: CpG Islands, and related genes, altered in the 27K and validated in 450K**

| **CpG Islands Name** | **Mean Diff Island 27k** | **SD Diff Island 27k** | **Mean Diff Island 450k** | **SD Diff Island 450k** | **Island Status 27k** | **Island Status 450k** | **HGNC** | **Description** |
| --- | --- | --- | --- | --- | --- | --- | --- | --- |
| chr17:48503056-48503887 | 0.056 | 0.039 | 0.064 | 0.045 | Hypermeth Supratentorial | Hypermeth Supratentorial | ACSF2 | Acyl-CoA Synthetase Family Member 2 |
| chr11:69468810-69469152 | 0.079 | 0.056 | 0.157 | 0.111 | Hypermeth Supratentorial | Hypermeth Supratentorial | CCND1 | Cyclin D1 |
| chr20:57581902-57582595 | 0.03 | 0.021 | 0.083 | 0.059 | Hypermeth Supratentorial | Hypermeth Supratentorial | CTSZ | Cathepsin Z |
| chr10:94820026-94823252 | -0.058 | 0.041 | -0.086 | 0.061 | Hypometh Supratentorial | Hypometh Supratentorial | CYP26C1 | Cytochrome P450 Family 26 Subfamily C Member 1 |
| chr10:135341255-135342561 | 0.057 | 0.04 | 0.055 | 0.039 | Hypermeth Supratentorial | Hypermeth Supratentorial | CYP2E1 | Cytochrome P450 Family 2 Subfamily E Member 1 |
| chr1:68512643-68513005 | -0.09 | 0.064 | -0.066 | 0.047 | Hypometh Supratentorial | Hypometh Supratentorial | DIRAS3 | Distinct Subgroup Of The Ras Family Member 3 |
| chr7:155246390-155251955 | -0.148 | 0.105 | -0.106 | 0.075 | Hypometh Supratentorial | Hypometh Supratentorial | EN2 | Engrailed Homeobox 2 |
| chr5:2748368-2757024 | 0.1 | 0.071 | 0.098 | 0.069 | Hypermeth Supratentorial | Hypermeth Supratentorial | IRX2 | Iroquois Homeobox 2 |
| chr11:2812415-2813396 | 0.038 | 0.027 | 0.089 | 0.063 | Hypermeth Supratentorial | Hypermeth Supratentorial | KCNQ1 | Potassium Voltage-Gated Channel Subfamily Q Member 1 |
| chr13:53313127-53314045 | 0.101 | 0.071 | 0.09 | 0.063 | Hypermeth Supratentorial | Hypermeth Supratentorial | LECT1 | Chondromodulin |
| chr7:130126017-130126801 | 0.06 | 0.043 | 0.11 | 0.078 | Hypermeth Supratentorial | Hypermeth Supratentorial | MEST | Mesoderm Specific Transcript |
| chr4:4864456-4864834 | 0.052 | 0.037 | 0.137 | 0.097 | Hypermeth Supratentorial | Hypermeth Supratentorial | MSX1 | Msh Homeobox 1 |
| chr11:6340445-6341909 | 0.049 | 0.035 | 0.192 | 0.136 | Hypermeth Supratentorial | Hypermeth Supratentorial | PRKCDBP | Protein Kinase C Delta Binding Protein |
| chr15:90039464-90039984 | -0.304 | 0.215 | -0.052 | 0.037 | Hypometh Supratentorial | Hypometh Supratentorial | RHCG | Rh Family C Glycoprotein |
| chr2:74667593-74669403 | 0.167 | 0.118 | 0.078 | 0.055 | Hypermeth Supratentorial | Hypermeth Supratentorial | RTKN | Rhotekin |
| chr20:42543097-42545137 | 0.153 | 0.108 | 0.077 | 0.055 | Hypermeth Supratentorial | Hypermeth Supratentorial | TOX2 | TOX High Mobility Group Box Family Member 2 |

**CpG Islands Name** = CpG island name (UCSC - hg19)

**Mean diff Island 27K** = Differential methylation value between Supratentorial/Infratentorial groups based on Illumina Infinium HumanMethylation27 Beadchip

**SD diff Island 27K** = Standard Deviation of the mean

**Mean diff Island 450K** = Differential methylation value between Supratentorial/Infratentorial groups based on 450K Beadchip

**SD diff Island 450K** = Standard Deviation of the mean

**Island status 27K** = Direction of methylation change for Supratentorial/Infratentorial groups relative to Illumina Infinium HumanMethylation27 Beadchip

**Island status 450K** = Direction of methylation change for Supratentorial/Infratentorial groups relative to 450K Beadchip

**HGNC** = HUGO Gene Nomenclature Committee
