# Supplementary material for: Integrated DNA methylation analysis identifies topographical and tumoral biomarkers in pilocytic astrocytomas
Source: Oncotarget. 2018 Feb 12;9(17):13807–21. doi: 10.18632/oncotarget.24480 (PMC5862617; doi:10.18632/oncotarget.24480)
Supplement: Supplementary file 3 [file oncotarget-09-13807-s003.docx]

**Supplementary Table 2: Average methylation value for each sample at CpG Islands, and related genes, altered in the 27K and validated in 450K**

| **TargetID** | **RefGene**  **Name** | **CpGIslands**  **Name** | **FCP1** | **FCP2** | **FCP3** | **FCP4** | **FCP5** | **FCP6** | **FCP7** | **FCP8** | **FCP9** | **FCP10** | **SVT1** | **SVT2** | **SVT3** | **SVT4** | **SVT5** | **SVT6** | **SVT7** | **SVT8** | **SVT9** | **SVT10** |
| --- | --- | --- | --- | --- | --- | --- | --- | --- | --- | --- | --- | --- | --- | --- | --- | --- | --- | --- | --- | --- | --- | --- |
| cg02516388 | ACSF2 | chr17:48503056-48503887 | 0.03 | 0.03 | 0.03 | 0.03 | 0.03 | 0.03 | 0.03 | 0.02 | 0.03 | 0.02 | 0.03 | 0.03 | 0.02 | 0.02 | 0.02 | 0.02 | 0.02 | 0.03 | 0.02 | 0.03 |
| cg14672994 | ACSF2 | chr17:48503056-48503887 | 0.12 | 0.19 | 0.15 | 0.06 | 0.06 | 0.08 | 0.09 | 0.38 | 0.10 | 0.17 | 0.07 | 0.42 | 0.38 | 0.22 | 0.35 | 0.25 | 0.22 | 0.20 | 0.30 | 0.15 |
| cg00953256 | CCND1 | chr11:69468810-69469152 | 0.34 | 0.60 | 0.64 | 0.34 | 0.18 | 0.42 | 0.61 | 0.73 | 0.51 | 0.52 | 0.70 | 0.53 | 0.43 | 0.60 | 0.61 | 0.53 | 0.47 | 0.85 | 0.35 | 0.56 |
| cg02723533 | CCND1 | chr11:69468810-69469152 | 0.53 | 0.84 | 0.84 | 0.54 | 0.22 | 0.58 | 0.80 | 0.94 | 0.90 | 0.69 | 0.96 | 0.79 | 0.56 | 0.96 | 0.90 | 0.87 | 0.57 | 0.96 | 0.73 | 0.84 |
| cg04717045 | CCND1 | chr11:69468810-69469152 | 0.39 | 0.69 | 0.70 | 0.46 | 0.15 | 0.44 | 0.70 | 0.81 | 0.75 | 0.57 | 0.76 | 0.64 | 0.38 | 0.76 | 0.70 | 0.50 | 0.40 | 0.88 | 0.47 | 0.55 |
| cg07426960 | CCND1 | chr11:69468810-69469152 | 0.13 | 0.30 | 0.41 | 0.18 | 0.10 | 0.22 | 0.36 | 0.35 | 0.34 | 0.29 | 0.49 | 0.28 | 0.24 | 0.38 | 0.31 | 0.47 | 0.28 | 0.61 | 0.17 | 0.33 |
| cg11802013 | CCND1 | chr11:69468810-69469152 | 0.53 | 0.74 | 0.69 | 0.56 | 0.37 | 0.60 | 0.72 | 0.81 | 0.77 | 0.64 | 0.78 | 0.78 | 0.61 | 0.74 | 0.66 | 0.73 | 0.60 | 0.84 | 0.56 | 0.78 |
| cg01623438 | CTSZ | chr20:57581902-57582595 | 0.62 | 0.49 | 0.43 | 0.60 | 0.75 | 0.57 | 0.47 | 0.66 | 0.70 | 0.56 | 0.39 | 0.53 | 0.63 | 0.62 | 0.62 | 0.58 | 0.60 | 0.18 | 0.71 | 0.61 |
| cg01663968 | CTSZ | chr20:57581902-57582595 | 0.09 | 0.07 | 0.10 | 0.06 | 0.07 | 0.08 | 0.08 | 0.05 | 0.08 | 0.06 | 0.08 | 0.07 | 0.20 | 0.07 | 0.04 | 0.09 | 0.05 | 0.06 | 0.06 | 0.08 |
| cg06385087 | CTSZ | chr20:57581902-57582595 | 0.14 | 0.02 | 0.11 | 0.02 | 0.02 | 0.02 | 0.03 | 0.03 | 0.05 | 0.12 | 0.13 | 0.03 | 0.25 | 0.15 | 0.09 | 0.15 | 0.29 | 0.03 | 0.10 | 0.19 |
| cg13021192 | CTSZ | chr20:57581902-57582595 | 0.07 | 0.09 | 0.07 | 0.08 | 0.05 | 0.10 | 0.11 | 0.06 | 0.06 | 0.10 | 0.08 | 0.08 | 0.08 | 0.10 | 0.06 | 0.08 | 0.11 | 0.06 | 0.07 | 0.14 |
| cg16179125 | CTSZ | chr20:57581902-57582595 | 0.07 | 0.03 | 0.05 | 0.04 | 0.03 | 0.03 | 0.04 | 0.03 | 0.07 | 0.04 | 0.05 | 0.04 | 0.26 | 0.29 | 0.05 | 0.16 | 0.07 | 0.04 | 0.07 | 0.05 |
| cg23265096 | CTSZ | chr20:57581902-57582595 | 0.50 | 0.47 | 0.38 | 0.34 | 0.54 | 0.36 | 0.31 | 0.48 | 0.53 | 0.48 | 0.34 | 0.56 | 0.53 | 0.48 | 0.55 | 0.43 | 0.54 | 0.37 | 0.55 | 0.49 |
| cg23679724 | CTSZ | chr20:57581902-57582595 | 0.74 | 0.71 | 0.63 | 0.70 | 0.79 | 0.56 | 0.65 | 0.75 | 0.69 | 0.68 | 0.65 | 0.72 | 0.75 | 0.75 | 0.73 | 0.69 | 0.74 | 0.72 | 0.80 | 0.74 |
| cg20322977 | CYP26C1 | chr10:94820026-94823252 | 0.70 | 0.45 | 0.41 | 0.26 | 0.79 | 0.48 | 0.60 | 0.53 | 0.59 | 0.45 | 0.42 | 0.49 | 0.51 | 0.29 | 0.38 | 0.38 | 0.47 | 0.38 | 0.65 | 0.45 |
| cg26404725 | CYP26C1 | chr10:94820026-94823252 | 0.69 | 0.78 | 0.82 | 0.87 | 0.89 | 0.73 | 0.85 | 0.81 | 0.78 | 0.67 | 0.80 | 0.89 | 0.76 | 0.59 | 0.75 | 0.78 | 0.68 | 0.82 | 0.65 | 0.88 |
| cg00436603 | CYP2E1 | chr10:135341255-135342561 | 0.39 | 0.49 | 0.58 | 0.33 | 0.81 | 0.54 | 0.41 | 0.22 | 0.50 | 0.55 | 0.61 | 0.55 | 0.57 | 0.50 | 0.48 | 0.62 | 0.45 | 0.76 | 0.18 | 0.66 |
| cg13315147 | CYP2E1 | chr10:135341255-135342561 | 0.03 | 0.33 | 0.03 | 0.04 | 0.03 | 0.04 | 0.09 | 0.04 | 0.26 | 0.03 | 0.05 | 0.51 | 0.20 | 0.04 | 0.09 | 0.04 | 0.20 | 0.21 | 0.03 | 0.11 |
| cg03641225 | DIRAS3 | chr1:68512643-68513005 | 0.56 | 0.52 | 0.46 | 0.27 | 0.80 | 0.45 | 0.42 | 0.55 | 0.37 | 0.42 | 0.32 | 0.35 | 0.40 | 0.38 | 0.42 | 0.44 | 0.37 | 0.38 | 0.57 | 0.18 |
| cg09118625 | DIRAS3 | chr1:68512643-68513005 | 0.76 | 0.71 | 0.68 | 0.81 | 0.57 | 0.73 | 0.70 | 0.89 | 0.71 | 0.74 | 0.68 | 0.60 | 0.60 | 0.65 | 0.63 | 0.72 | 0.62 | 0.56 | 0.54 | 0.68 |
| cg13697378 | DIRAS3 | chr1:68512643-68513005 | 0.61 | 0.63 | 0.66 | 0.74 | 0.53 | 0.77 | 0.64 | 0.84 | 0.66 | 0.63 | 0.64 | 0.58 | 0.57 | 0.54 | 0.58 | 0.63 | 0.58 | 0.57 | 0.56 | 0.56 |
| cg21808053 | DIRAS3 | chr1:68512643-68513005 | 0.53 | 0.50 | 0.44 | 0.26 | 0.44 | 0.33 | 0.50 | 0.81 | 0.36 | 0.43 | 0.51 | 0.44 | 0.44 | 0.44 | 0.45 | 0.41 | 0.40 | 0.44 | 0.43 | 0.37 |
| cg22901840 | DIRAS3 | chr1:68512643-68513005 | 0.49 | 0.54 | 0.62 | 0.79 | 0.51 | 0.80 | 0.55 | 0.56 | 0.52 | 0.56 | 0.55 | 0.46 | 0.51 | 0.58 | 0.51 | 0.54 | 0.51 | 0.50 | 0.49 | 0.47 |
| cg24871743 | DIRAS3 | chr1:68512643-68513005 | 0.58 | 0.61 | 0.58 | 0.63 | 0.34 | 0.67 | 0.58 | 0.67 | 0.58 | 0.54 | 0.59 | 0.41 | 0.33 | 0.46 | 0.59 | 0.48 | 0.44 | 0.35 | 0.32 | 0.42 |
| cg11648289 | EN2 | chr7:155246390-155251955 | 0.02 | 0.03 | 0.03 | 0.03 | 0.03 | 0.03 | 0.04 | 0.02 | 0.03 | 0.03 | 0.03 | 0.02 | 0.03 | 0.02 | 0.03 | 0.03 | 0.03 | 0.03 | 0.02 | 0.03 |
| cg21051046 | EN2 | chr7:155246390-155251955 | 0.61 | 0.64 | 0.25 | 0.14 | 0.80 | 0.66 | 0.55 | 0.84 | 0.53 | 0.24 | 0.15 | 0.53 | 0.14 | 0.18 | 0.40 | 0.31 | 0.13 | 0.20 | 0.10 | 0.17 |
| cg26504021 | IRX2 | chr5:2748368-2757024 | 0.42 | 0.21 | 0.22 | 0.10 | 0.06 | 0.25 | 0.14 | 0.31 | 0.23 | 0.10 | 0.13 | 0.22 | 0.57 | 0.07 | 0.33 | 0.23 | 0.46 | 0.31 | 0.38 | 0.34 |
| cg06233503 | KCNQ1 | chr11:2812415-2813396 | 0.46 | 0.16 | 0.33 | 0.62 | 0.34 | 0.25 | 0.26 | 0.13 | 0.30 | 0.14 | 0.24 | 0.55 | 0.25 | 0.27 | 0.29 | 0.19 | 0.29 | 0.19 | 0.52 | 0.39 |
| cg17820828 | KCNQ1 | chr11:2812415-2813396 | 0.25 | 0.41 | 0.57 | 0.26 | 0.24 | 0.27 | 0.37 | 0.20 | 0.34 | 0.46 | 0.58 | 0.36 | 0.43 | 0.34 | 0.50 | 0.44 | 0.55 | 0.66 | 0.29 | 0.42 |
| cg19728223 | KCNQ1 | chr11:2812415-2813396 | 0.10 | 0.13 | 0.39 | 0.51 | 0.04 | 0.06 | 0.14 | 0.09 | 0.20 | 0.17 | 0.17 | 0.09 | 0.23 | 0.24 | 0.13 | 0.10 | 0.31 | 0.10 | 0.10 | 0.12 |
| cg00708598 | LECT1 | chr13:53313127-53314045 | 0.08 | 0.11 | 0.18 | 0.10 | 0.08 | 0.10 | 0.16 | 0.08 | 0.09 | 0.11 | 0.16 | 0.17 | 0.22 | 0.08 | 0.21 | 0.15 | 0.30 | 0.22 | 0.13 | 0.11 |
| cg22398616 | LECT1 | chr13:53313127-53314045 | 0.18 | 0.29 | 0.35 | 0.28 | 0.16 | 0.30 | 0.36 | 0.17 | 0.09 | 0.31 | 0.55 | 0.53 | 0.29 | 0.11 | 0.45 | 0.34 | 0.46 | 0.64 | 0.13 | 0.32 |
| cg01888566 | MEST | chr7:130126017-130126801 | 0.33 | 0.29 | 0.50 | 0.07 | 0.13 | 0.09 | 0.19 | 0.32 | 0.25 | 0.17 | 0.22 | 0.14 | 0.42 | 0.19 | 0.45 | 0.24 | 0.48 | 0.35 | 0.59 | 0.26 |
| cg02490034 | MEST | chr7:130126017-130126801 | 0.70 | 0.60 | 0.47 | 0.68 | 0.68 | 0.49 | 0.48 | 0.72 | 0.74 | 0.60 | 0.43 | 0.69 | 0.63 | 0.59 | 0.68 | 0.64 | 0.61 | 0.28 | 0.80 | 0.65 |
| cg08077673 | MEST | chr7:130126017-130126801 | 0.52 | 0.26 | 0.39 | 0.12 | 0.56 | 0.19 | 0.35 | 0.33 | 0.22 | 0.21 | 0.29 | 0.16 | 0.52 | 0.17 | 0.35 | 0.37 | 0.49 | 0.21 | 0.63 | 0.31 |
| cg09059945 | MEST | chr7:130126017-130126801 | 0.33 | 0.18 | 0.46 | 0.09 | 0.15 | 0.10 | 0.41 | 0.14 | 0.17 | 0.13 | 0.23 | 0.13 | 0.21 | 0.14 | 0.29 | 0.32 | 0.62 | 0.29 | 0.25 | 0.42 |
| cg09872616 | MEST | chr7:130126017-130126801 | 0.19 | 0.17 | 0.48 | 0.14 | 0.10 | 0.08 | 0.39 | 0.23 | 0.25 | 0.15 | 0.35 | 0.10 | 0.40 | 0.20 | 0.49 | 0.41 | 0.43 | 0.33 | 0.45 | 0.45 |
| cg15164103 | MEST | chr7:130126017-130126801 | 0.45 | 0.50 | 0.39 | 0.51 | 0.26 | 0.37 | 0.30 | 0.76 | 0.30 | 0.46 | 0.31 | 0.34 | 0.49 | 0.46 | 0.62 | 0.40 | 0.51 | 0.21 | 0.71 | 0.48 |
| cg01785568 | MSX1 | chr4:4864456-4864834 | 0.79 | 0.47 | 0.64 | 0.77 | 0.16 | 0.23 | 0.58 | 0.53 | 0.56 | 0.45 | 0.48 | 0.19 | 0.74 | 0.69 | 0.78 | 0.72 | 0.64 | 0.48 | 0.88 | 0.78 |
| cg03199651 | MSX1 | chr4:4864456-4864834 | 0.31 | 0.07 | 0.12 | 0.05 | 0.05 | 0.06 | 0.07 | 0.06 | 0.05 | 0.07 | 0.06 | 0.14 | 0.07 | 0.08 | 0.05 | 0.06 | 0.06 | 0.06 | 0.05 | 0.05 |
| cg03843978 | MSX1 | chr4:4864456-4864834 | 0.53 | 0.47 | 0.54 | 0.61 | 0.22 | 0.28 | 0.55 | 0.38 | 0.55 | 0.50 | 0.48 | 0.27 | 0.59 | 0.54 | 0.63 | 0.56 | 0.57 | 0.48 | 0.70 | 0.56 |
| cg09573795 | MSX1 | chr4:4864456-4864834 | 0.86 | 0.67 | 0.73 | 0.81 | 0.61 | 0.71 | 0.81 | 0.78 | 0.77 | 0.72 | 0.68 | 0.56 | 0.81 | 0.79 | 0.77 | 0.75 | 0.77 | 0.55 | 0.87 | 0.80 |
| cg09748975 | MSX1 | chr4:4864456-4864834 | 0.92 | 0.61 | 0.79 | 0.85 | 0.23 | 0.28 | 0.75 | 0.78 | 0.86 | 0.68 | 0.63 | 0.28 | 0.87 | 0.79 | 0.90 | 0.81 | 0.82 | 0.64 | 0.94 | 0.91 |
| cg14167596 | MSX1 | chr4:4864456-4864834 | 0.34 | 0.06 | 0.14 | 0.07 | 0.05 | 0.08 | 0.09 | 0.06 | 0.05 | 0.07 | 0.06 | 0.05 | 0.08 | 0.10 | 0.06 | 0.04 | 0.06 | 0.06 | 0.04 | 0.07 |
| cg20161179 | MSX1 | chr4:4864456-4864834 | 0.44 | 0.06 | 0.27 | 0.10 | 0.07 | 0.07 | 0.08 | 0.07 | 0.06 | 0.09 | 0.05 | 0.05 | 0.32 | 0.16 | 0.28 | 0.15 | 0.23 | 0.14 | 0.20 | 0.11 |
| cg20891301 | MSX1 | chr4:4864456-4864834 | 0.83 | 0.63 | 0.73 | 0.80 | 0.32 | 0.39 | 0.74 | 0.66 | 0.69 | 0.66 | 0.60 | 0.35 | 0.84 | 0.69 | 0.83 | 0.77 | 0.75 | 0.58 | 0.89 | 0.82 |
| cg22609784 | MSX1 | chr4:4864456-4864834 | 0.67 | 0.46 | 0.50 | 0.65 | 0.15 | 0.30 | 0.53 | 0.56 | 0.38 | 0.33 | 0.39 | 0.27 | 0.66 | 0.50 | 0.65 | 0.59 | 0.51 | 0.32 | 0.76 | 0.59 |
| cg24840099 | MSX1 | chr4:4864456-4864834 | 0.76 | 0.36 | 0.59 | 0.72 | 0.15 | 0.25 | 0.62 | 0.39 | 0.65 | 0.49 | 0.41 | 0.21 | 0.77 | 0.64 | 0.76 | 0.65 | 0.64 | 0.45 | 0.84 | 0.69 |
| cg27038439 | MSX1 | chr4:4864456-4864834 | 0.93 | 0.86 | 0.88 | 0.89 | 0.33 | 0.49 | 0.88 | 0.79 | 0.91 | 0.84 | 0.76 | 0.79 | 0.93 | 0.82 | 0.93 | 0.89 | 0.85 | 0.79 | 0.95 | 0.93 |
| cg05628549 | PRKCDBP | chr11:6340445-6341909 | 0.39 | 0.28 | 0.24 | 0.41 | 0.17 | 0.34 | 0.12 | 0.08 | 0.09 | 0.09 | 0.29 | 0.37 | 0.15 | 0.30 | 0.38 | 0.18 | 0.45 | 0.32 | 0.48 | 0.22 |
| cg16245261 | PRKCDBP | chr11:6340445-6341909 | 0.37 | 0.18 | 0.19 | 0.55 | 0.41 | 0.34 | 0.17 | 0.10 | 0.27 | 0.26 | 0.16 | 0.23 | 0.08 | 0.50 | 0.11 | 0.07 | 0.33 | 0.13 | 0.23 | 0.26 |
| cg18392783 | PRKCDBP | chr11:6340445-6341909 | 0.27 | 0.33 | 0.28 | 0.12 | 0.17 | 0.15 | 0.16 | 0.37 | 0.13 | 0.13 | 0.12 | 0.19 | 0.47 | 0.18 | 0.49 | 0.26 | 0.44 | 0.30 | 0.30 | 0.30 |
| cg18959478 | PRKCDBP | chr11:6340445-6341909 | 0.42 | 0.44 | 0.41 | 0.17 | 0.33 | 0.28 | 0.23 | 0.47 | 0.13 | 0.16 | 0.21 | 0.31 | 0.54 | 0.21 | 0.62 | 0.36 | 0.44 | 0.32 | 0.41 | 0.45 |
| cg10453365 | RHCG | chr15:90039464-90039984 | 0.54 | 0.50 | 0.39 | 0.41 | 0.34 | 0.04 | 0.51 | 0.57 | 0.41 | 0.49 | 0.38 | 0.51 | 0.05 | 0.14 | 0.03 | 0.04 | 0.12 | 0.05 | 0.03 | 0.02 |
| cg24653181 | RHCG | chr15:90039464-90039984 | 0.57 | 0.57 | 0.49 | 0.54 | 0.57 | 0.12 | 0.58 | 0.61 | 0.51 | 0.63 | 0.38 | 0.62 | 0.17 | 0.28 | 0.06 | 0.06 | 0.21 | 0.06 | 0.06 | 0.05 |
| cg00689340 | RTKN | chr2:74667593-74669403 | 0.12 | 0.25 | 0.54 | 0.21 | 0.33 | 0.17 | 0.36 | 0.54 | 0.35 | 0.39 | 0.58 | 0.47 | 0.55 | 0.27 | 0.52 | 0.46 | 0.41 | 0.65 | 0.56 | 0.48 |
| cg04369341 | TOX2 | chr20:42543097-42545137 | 0.14 | 0.15 | 0.49 | 0.16 | 0.07 | 0.11 | 0.20 | 0.36 | 0.29 | 0.38 | 0.23 | 0.57 | 0.62 | 0.19 | 0.24 | 0.39 | 0.63 | 0.44 | 0.57 | 0.30 |
| cg11319389 | TOX2 | chr20:42543097-42545137 | 0.07 | 0.08 | 0.37 | 0.08 | 0.06 | 0.08 | 0.11 | 0.16 | 0.11 | 0.20 | 0.15 | 0.43 | 0.48 | 0.08 | 0.11 | 0.21 | 0.31 | 0.30 | 0.36 | 0.10 |
