# Supplementary material for: Integrated DNA methylation analysis identifies topographical and tumoral biomarkers in pilocytic astrocytomas
Source: Oncotarget. 2018 Feb 12;9(17):13807–21. doi: 10.18632/oncotarget.24480 (PMC5862617; doi:10.18632/oncotarget.24480)
Supplement: Supplementary file 4 [file oncotarget-09-13807-s004.docx]

**Supplementary Table 3: Sequences of primers used for qRT-PCR**

| **Gene** | **Primer orientation** | **Nucleotide sequence** | **Amplicon (bp)** |
| --- | --- | --- | --- |
| ***EN2*** | Forward | 5-GGCGTGGGTCTACTGTACG-3 | 161 |
|  | Reverse | 5-ACCTGTTGGTCTGGAACTCG-3 |  |
| ***IRX2*** | Forward | 5-GGCTTCCCGTCCTACATG-3 | 80 |
|  | Reverse | 5-CCGTACGGGTGGTAGCTGAT-3 |  |
| ***TOX2*** | Forward | 5-TGGTGTCCAACATGCTAGCA-3 | 100 |
|  | Reverse | 5-CCCGAGTCATAGGCAGCC-3 |  |
| ***TFRC*** | Forward | 5-GGCACAGCTCTCCTATTGA-3 | 112 |
|  | Reverse | 5-CAAAGTCTCCAGCACTCCA-3 |  |
